# Simultaneous Measurement of Contraction and Calcium Transients in Stem Cell Derived Cardiomyocytes

**DOI:** 10.1007/s10439-017-1933-2

**Published:** 2017-10-03

**Authors:** A. Ahola, R.-P. Pölönen, K. Aalto-Setälä, J. Hyttinen

**Affiliations:** 1 BioMediTech Institute and Faculty of Biomedical Sciences and Engineering, Tampere University of Technology, Korkeakoulunkatu 10, 33720 Tampere, Finland; 2 BioMediTech Institute and Faculty of Medicine and Life Sciences, University of Tampere, Tampere, Finland; 3 Heart Hospital, Tampere University Hospital, Tampere, Finland

**Keywords:** Biomechanics, Fluorescence, Motion analysis, Calcium-contraction coupling

## Abstract

Induced pluripotent stem cell derived cardiomyocytes (iPSC-CM) provide a powerful platform for disease modeling and drug development *in vitro*. Traditionally, electrophysiological methods or fluorescent dyes (e.g. calcium) have been used in their functional characterization. Recently, video microscopy has enabled non-invasive analysis of CM contractile motion. Simultaneous assessments of motion and calcium transients have not been generally conducted, as motion detection methods are affected by changing pixel intensities in calcium imaging. Here, we present for the first time a protocol for simultaneous video-based measurement of contraction and calcium with fluorescent dye Fluo-4 videos without corrections, providing data on both ionic and mechanic activity. The method and its accuracy are assessed by measuring the effect of fluorescence and background light on transient widths and contraction velocity amplitudes. We demonstrate the method by showing the contraction-calcium relation and measuring the transient time intervals in catecholaminergic polymorphic ventricular tachycardia patient specific iPSC-CMs and healthy controls. Our validation shows that the simultaneous method provides comparable data to combined individual measurements, providing a new tool for measuring CM biomechanics and calcium simultaneously. Our results with calcium sensitive dyes suggest the method could be expanded to use with other fluorescent reporters as well.

**Electronic supplementary material:**

The online version of this article (doi:10.1007/s10439-017-1933-2) contains supplementary material, which is available to authorized users.

## Introduction

Cardiovascular diseases represent the leading cause of death globally. Additionally, adverse effects of cardioactive and non-cardiac drugs remain the most common reason for market withdrawal. Induced pluripotent stem cell derived cardiomyocytes (iPSC-CM) have presented a promising platform for both disease modeling and drug development with healthy and patient specific cells. These studies have been traditionally done using electrical measurements, while methods for measuring mechanical function of cells without extensive instrumentation have only recently been emerging. High throughput methods for drug screening with iPSC-CMs are needed, as the large variability in electrophysiological results regarding iPSC-CM bring about the need for a greater number of experiments.^15^ These methods could be used in addition for development of personalized therapy for genetic cardiac diseases, such as catecholaminergic polymorphic ventricular tachycardia (CPVT).^10^

The commonly used methods for analyzing electrophysiological functions of cardiomyocytes (CM) include calcium imaging,^18^ patch clamping^8^,^11^ and microelectrode array^11^ studies. However, these measurements often require manual operation by trained personnel and thus are not well suited towards high-throughput applications. In addition, while the ionic function they measure control the mechanical function of the CMs, the resulting signals do not relate directly to the resulting biomechanics that is the actual CM functional aspect. CM biomechanics has traditionally been measured *in vitro* using atomic force microscopy,^19^ mechanical transduction with microposts^4^,^22^ and traction force microscopy.^21^ Video-based methods have been developed and studied by us^1^,^2^,^9^ and by several other groups.^6^,^7^,^14^ As the method is non-invasive and has easy to use instrumentation, video analysis has good high-throughput capabilities.

Calcium imaging can be used as a powerful tool for high throughput profiling of drug responses *in vitro*. In CMs, calcium plays a key role as a second messenger of excitation contraction coupling. Drug effects are reflected as changes in calcium transient profile. With fluorescent calcium reporter dyes, either small molecule or genetically encoded, it is possible to visualize and record calcium fluxes inside CMs. Special high throughput imaging apparatuses, for example Hamamatsu FDSS,^3^ allow simultaneous reading of calcium transients from up to hundreds of samples. These platforms can be used to study several disease models and drugs in a short time period compared to conventional microscopes. High content screening produces large amount of data, which require automated software for handling and analyzing the massive amounts of data.^17^

The major benefit of human iPSC (hiPSC) in CM studies is their power to provide CMs from specific phenotypes. Here CPVT cells was selected as targeted application example for our novel methodology. CPVT is a rare but severe inherited cardiac disease in structurally normal hearts, which may lead to sudden cardiac death. CPVT patients experience arrhythmias during mental or physical stress. CPVT is associated with mutations in *RYR2* gene, coding for cardiac ryanodine receptor (RYR), among few other disease-associated genes. Therapy includes drug treatment with beta-blockers, implantable cardiac defibrillators or left cardiac sympathetic denervation.^23^ The calcium abnormalities arising from mutations in RYR2 reflect to contractile motion of CMs. Therefore, CPVT specific iPSC-CMs are interesting platform to study not only calcium kinetics but also the contractile motion simultaneously.

So far, the ionic and mechanical functions have been studied simultaneously using specific platforms, such as on microposts.^4^,^22^ Video-based measurements, however, do not require such systems: microscopes and video cameras are typical laboratory instruments, which as such enable video-based measurements. Methods to provide combined ionic and biomechanic assessment of the CMs could offer novel avenues to look into disease mechanisms and drug effects. Previously, it has not been able to track motion in bright field microscopy simultaneously with fluorescent reporters due to the effects of fluorescent induced changes in pixel intensity on the motion detection methods. In previous studies the fluorescence background has been subtracted from images with mixed phase-contrast and fluorescence.^6^ In another study, calcium reporter GCaMP6f –gene was used and simultaneous measurement was conducted with computed local intensity normalization in each interrogation window to counteract the effect of the intensity changes.^7^

In this study, we combine concurrent calcium imaging with our previously developed video analysis method, which uses minimum quadratic difference (MQD) based particle image velocimetry (PIV) and assess biomechanical and ionic function simultaneously from videos of fluorescent calcium imaging. As in the MQD method we used^1^ sets equal weight to all image pixels,^5^ we hypothesized that the method could work also in calcium imaging by increasing background lighting conditions to make motion more visible. To verify our hypothesis, consecutive frames with fluorescent calcium dye excitation and transmission channels were measured with two background light levels. We validated the method by comparing contraction and calcium transient widths as well as PIV amplitudes from frames with and without the fluorescent excitation, and with and without background light. We demonstrated the method by measuring the relation of contraction and calcium in wild type (WT) and CPVT cells, and calculated the time interval between the maximum signal onset and offset rates of change in both signals.

## Materials and Methods

### Patient-Specific Human iPSC Lines

Details on hiPSC culturing and CM differentiation are available in supplements.

In this study both normal control CMs and diseased CMs prone to arrhythmias were used. The diseased CPVT cells were derived from patients having mutations in *RYR2* gene, associated with structural changes in RYR, resulting in abnormal release of Ca^2+^ from sarcoplasmic reticulum and increased ventricular arrhythmias when heart rate increases. Two CPVT patient specific hiPSC lines were used: UTA.05605.CPVT carrying exon 3 deletion and UTA.05404.CPVT carrying V4563F mutation in *RYR2* gene (referred to as CPVTa and CPVTb respectively). UTA.04602.WT cell line was used as a control. The collection of biopsies for generating patient specific hiPSC lines was approved by the ethical committee of Pirkanmaa Hospital District (Aalto-Setälä R08070) and written informed consent was obtained from all the donors.

### Imaging Protocol

Details on imaging protocol are available in supplements.

**Figure 1:**
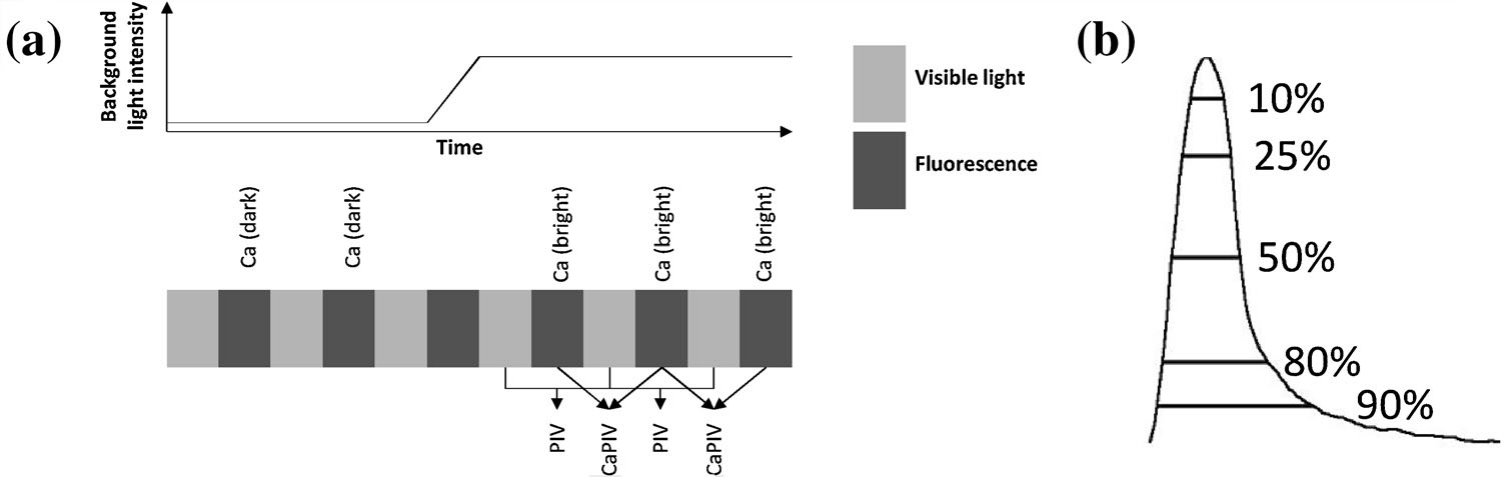
Imaging protocol, processing of the video channels and definition of transient duration parameters. (**a**) Fluorescent calcium and visible light were recorded in consecutive frames. Midway during the recording, light source was turned on to increase the background light intensity, allowing video signal to be captured. Traditional PIV was measured from the frames with only visible light and CaPIV signal was measured from frames with calcium fluorescence present. Calcium signal was categorized based on the background light intensity to Ca (dark) and Ca (bright). (**b**) Transient duration parameters are defined as percentages from the peak maximum, similarly as when classifying action potentials.

CMs plated on glass coverslips were loaded with Fluo-4 AM. Imaging was done using Olympus IX71 inverted microscope with Polychrome V and TH4-200 light sources and Andor iXon 885 EMCCD camera. Cells were perfused with warmed (37 °C) perfusate medium. Two channels were recorded consecutively: the Olympus light source was turned on to obtain video image while still being able to discern Ca^2+^ fluctuations. The recording process is illustrated in Fig. 1a. In total, 25 videos were recorded: 7 CPVTa, 5 CPVTb and 13 WT videos. The recordings were all baseline measurements.

### Video Data Processing

The obtained recordings, consisting of video imaging and calcium imaging data with light source off and on, were categorized and processed. The region of interest for each recording was selected based on both intensity of the calcium fluctuations and observed motion.

MQD based PIV, as described previously,^1^,^2^,^9^ was used for determining velocity vector fields in the video image series. The process was applied to both transmission and calcium imaging with background light on, resulting in two series of velocity vector fields from which motion signals are calculated: one from the frames with only visible light—traditional PIV measurement—and another from the frames with fluorescent emission of calcium indicator, defined as calcium PIV (CaPIV). Only the frames with light source on were included in these sets. Calcium signals were calculated from the pixel intensity in each frame. With respect to the light source status, two consecutive data series were obtained: calcium with fluorescent light from Polychrome V—Ca (dark)—and calcium with light from both Polychrome V and Olympus TH4-200 (transmission) - Ca (bright). With this method, four signal types were obtained—two from both modalities: calcium and motion. The process is illustrated in Fig. 1a.

Using our previously described method, shortly, the directional motion signals indicating contraction and relaxation velocities were obtained from velocity vector fields by determining a CM beating focus point. The motion directed towards it was defined as radial movement and the perpendicular motion as tangential, resulting in motion signals. As opposed to the previous article, each vector was divided in these components with respect to their location. The same region of interest was used for calcium imaging. Further, contraction signals were calculated from the motion signals by integration with respect to time. Linear detrending was applied to the resulting signals to correct the baseline.

As a non-ratiometric dye was used and absolute contraction amplitude was not assessed, calcium, contraction and motion signals were all normalized to interval [0,1] by linear rescaling for visualization. The motion signal baseline was then set to zero, with contraction being positive and relaxation negative. For calcium and contraction signals, one beat long cross-correlation based averaged signal templates were calculated, as described earlier.^2^ The averaging was synchronized based on the maximum rate of change during the onset of the contraction or transient. A correlation coefficient of 0.5 was used to exclude only the most abnormal beats.

### Signal Comparison

The similarity of the recorded signals was measured by determining parameters describing the waveform widths and amplitudes. The duration of the contraction in the templates was characterized by calculating contraction duration (CD) parameters and the duration of calcium by calcium transient duration (CTD) parameters. The durations at percentages of the maximum peak height were defined as shown in Fig. 1b—an approach similar to characterization of action potentials. The used parameters were CD10, CD25, CD50, CD80 and CD90, representing the width of the peak at each percentage measured from the peak in each waveform. For calcium, the same CTD parameters were determined, respectively.

Temporal differences between the contraction and calcium transient were measured using the combined signals from the maximum rate of change in the onset and offset of CaPIV and Ca (bright). As the calcium transients may have several peaks of different heights, this approach was selected instead of comparing the time difference between peaks themselves.

### Statistics

Mean values and standard deviations of CDs and CTDs, as well as temporal differences between contraction and calcium were calculated. CDs and CTDs were tested against each other using a two-sample *t* test with all combinations of PIV, CaPIV, Ca (dark) and Ca (bright). In addition, durations from WT cell videos (*n* = 13) were tested against those measured from CPVT cell videos (CPVTa *n* = 7, CPVTb *n* = 5) regarding all combinations of signal types from the same modality—calcium and motion. Waveform amplitude similarity was determined by calculating the symmetric mean absolute percentage error of contraction motion velocity magnitudes of each contraction in PIV and CaPIV signals. Linear regression was used to map width parameters against the other signal types, and their similarity was estimated using linear regression *R* ^2^ parameter. *P*-values below 0.05 were considered statistically significant. The statistical analysis was performed in MATLAB R2014a, The Mathworks, Inc.

## Results

### Beating Characteristics

For WT, the average beating rate was 22 beats per min with a ± 10.8 standard deviation. For CPVTa and CPVTb the values were 20.7 ± 12.9 and 12.2 ± 5.6 beats per min, respectively. The number of beats in CPVT videos were lower than in WT videos as some videos only had two beats with the background light on/off.

### Representative Signals

**Figure 2:**
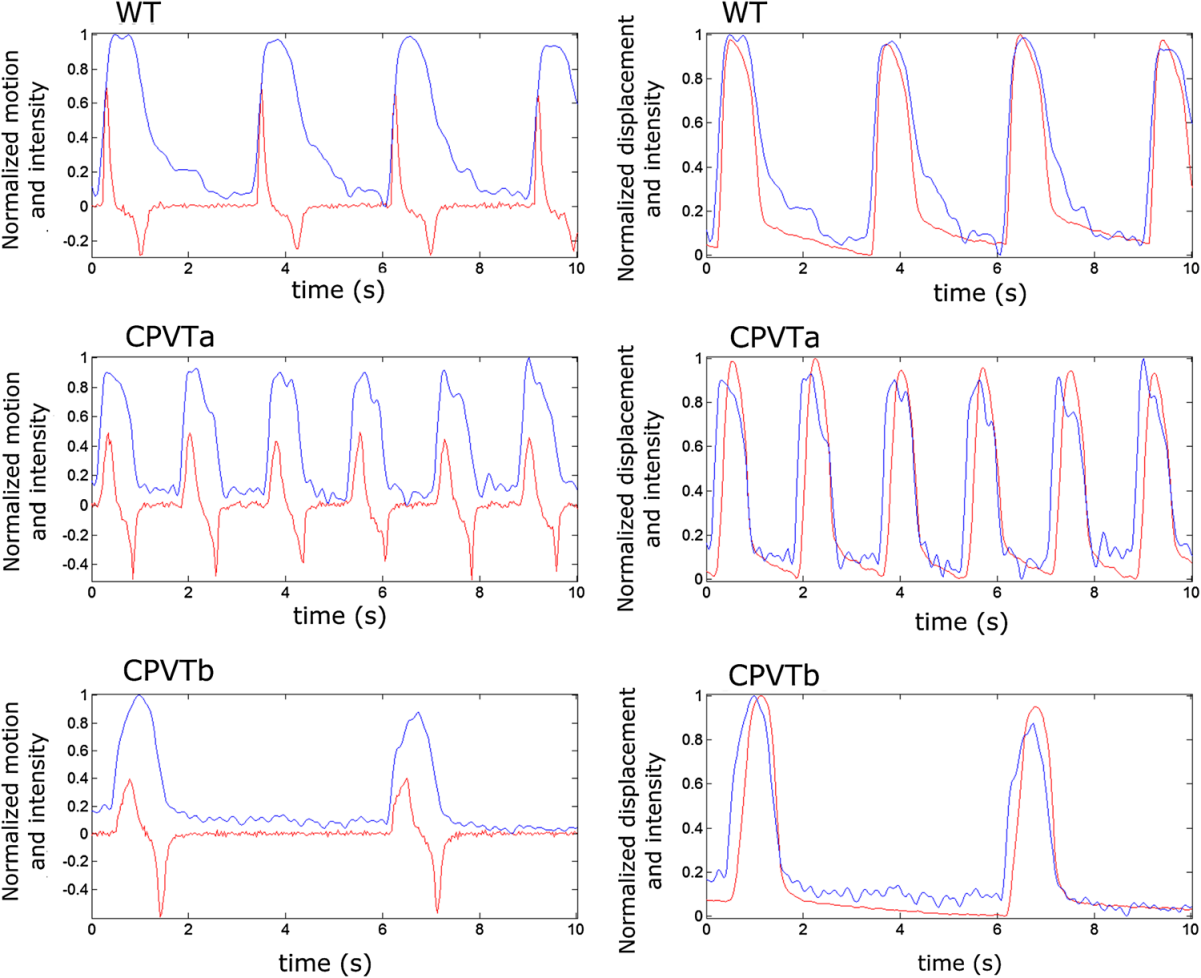
Representative motion (left side) and displacement (right side) signals plotted over calcium transients for the three cell lines. In the motion signals, the contractile movement velocity is represented with and upward peak and the relaxation movement velocity as a downward peak in red, while calcium transient is shown in blue. In the displacement signals (right side), representing the motion velocity signal integral with respect to time, contraction is shown in red and calcium transient in blue. Representative signals are shown for the three cell lines.

Representative signals with synchronized motion (CaPIV) and calcium signals (Ca (bright)) were calculated. For each cell line, both motion and contraction signals are shown with overlaid calcium transients in Fig. 2. In motion signals (left side), an upward peak signifies contractile and downward peak relaxation movement velocity. Motion signal is used for illustrating the fine details of the movement. Contraction signal (right side)—the integral of the motion signal with respect to time—shows displacement during a contraction. The contraction signal enables easier comparison of calcium and contraction. For this illustration, the signals were linearly normalized separately for visualization.

In the visual inspection of the displacement and calcium signals from studied WT cell populations, contraction started only after increase in calcium transient had started. Contraction signals typically had a steeper offset slope than calcium, which had a longer tail, and started to fall slightly earlier. In CPVTa cells, in some signals motion followed the calcium transient with a longer delay than in WT. No evident afterdepolarizations or tailing of calcium was present in the signals. In CPVTb, the contraction signal started to fall slightly later than the calcium transient.

### Method Validation

The simultaneous recording method was validated by comparing signals obtained from PIV (motion from videos without fluorescence), CaPIV (motion from videos with fluorescence), Ca (bright) (calcium with background light) and Ca (dark) (without background light) signals. CTD parameters from the two calcium signals, CD parameters from the two contraction signals, and amplitudes from the two motion velocity signals were calculated.

**Figure 3:**
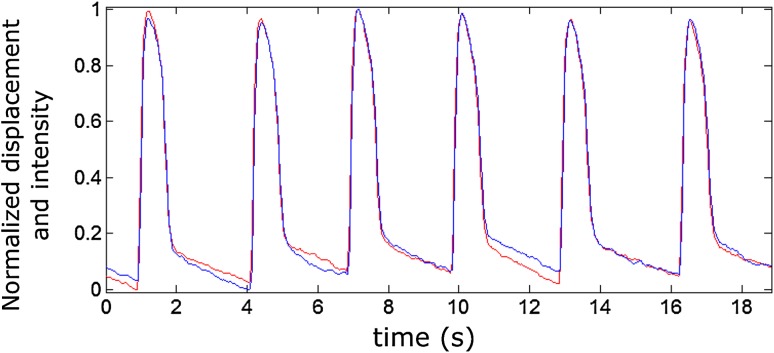
A representative displacement measurement showing overlaid CaPIV (blue) with fluorescence and PIV (shown in red) without fluorescence.

PIV and CaPIV did not show statistically significant differences between the means of CD parameters. This can be seen in Fig. 3, where the overlaid CaPIV (in blue) and PIV (red) show a very similar curve, with minor deviations showing near the baseline. Similarly, Ca (bright) and Ca (dark) did not exhibit significant differences in CTD parameters. This indicates that no major differences were induced by the change in lighting to enable the combined signal measurement.

The symmetric mean absolute percentage errors between PIV and CaPIV contraction velocity amplitudes were calculated for the contractions in each recording. The difference was found to be 6.42 ± 3.44% in WT, 4.11 ± 5.01% in CPVTa and 2.73 ± 1.57% in CPVTb. The total error for all recordings was 5.04 ± 3.87%.

### Assessment of Cell Line Functions Using Simultaneous Contraction and Calcium

#### Contraction and Calcium Signal Characteristics

The widths of the contraction peak in CaPIV and the calcium transient Ca (bright) in the simultaneous measurement were characterized by parameters indicating their duration at percentages of the peak maximum height. The CD- and CTD-parameters were calculated at signal levels 10, 25, 50, 80 and 90 and shown in Table 1.

**Table 1:** Calcium and contraction signal characterization.

	CD10	CD25	CD50	CD80	CD90	CTD10	CTD25	CTD50	CTD80	CTD90
WT	229 ± 77 ms	343 ± 118 ms	475 ± 165 ms	684 ± 206 ms	888 ± 391 ms	328 ± 104 ms	513 ± 148 ms	797 ± 227 ms	1315 ± 420 ms	1663 ± 570 ms
CPVTa	317 ± 185 ms	436 ± 200 ms	590 ± 266 ms	888 ± 250 ms	1340 ± 651 ms	356 ± 150 ms	581 ± 236 ms	797 ± 305 ms	1057 ± 330 ms	1283 ± 414 ms
CPVTb	255 ± 175 ms	379 ± 181 ms	515 ± 202 ms	714 ± 190 ms	919 ± 301 ms	273 ± 150 ms	413 ± 244 ms	589 ± 268 ms	788 ± 296 ms	905 ± 415 ms

The characterization of contraction and calcium in the simultaneous measurement. The parameters CD10–CD90 refer to contraction peak widths and CTD10–CTD90 to calcium transient widths at percentages of maximum peak height

In both CD and CTD parameters, the standard deviation was very high indicating high variance within the cell lines.

#### Similarity of Signals Between Modalities

Linear regressions between pairs of contraction/calcium transient durations for the four different signals were calculated. The results are shown in Table 2. Pairs of the same modality (leftmost tables) show high *R* ^2^ values: PIV exhibits very high coefficient of determination (*R* ^2^) values with CaPIV in both WT and CPVT cells. Only in CD90 the value of CPVTb *R* ^2^ was below 0.9. Likewise, high *R* ^2^ values were obtained from regression between Ca (bright) and Ca (dark) even though the signals were not concurrent like motion signals. In calcium, *R* ^2^ values below 0.9 were seen in CPVTa CTD25 and CTD90, and a relatively low value in CTD10 of CPVTb. Low *R* ^2^ values were typically seen in cross-modality pairs near the baseline (CD/CTD80 and CD/CTD90), especially in CPVTa. These results indicate the simultaneous measurement works well—the signals of the same modality are indeed very similar in shape. Further, the simultaneous motion and calcium measurements show a disconnection of calcium and motion in CPVT lines—especially CPVTa—which is not evident in calcium measurements.

**Table 2:** Linear regression of CD and CTD parameters.

	PIV/CaPIV			PIV/Ca (bright)			CaPIV/Ca (bright)		
	WT	CPVTa	CPVTb	WT	CPVTa	CPVTb	WT	CPVTa	CPVTb
10	0.983	0.997	0.975	0.663	0.128	0.430	0.698	0.136	0.511
25	0.943	0.997	0.996	0.737	0.540	0.897	0.698	0.537	0.888
50	0.965	0.997	0.995	0.637	0.731	0.732	0.612	0.684	0.752
80	0.967	0.991	0.976	0.640	0.129	0.451	0.630	0.091	0.384
90	0.966	0.989	0.817	0.710	0.000	0.186	0.724	0.003	0.057

	Ca (bright)/Ca (dark)			PIV/Ca (dark)			CaPIV/Ca (dark)		
	WT	CPVTa	CPVTb	WT	CPVTa	CPVTb	WT	CPVTa	CPVTb
10	0.914	0.913	0.567	0.518	0.138	0.958	0.525	0.150	0.945
25	0.939	0.753	0.859	0.685	0.605	0.834	0.612	0.616	0.793
50	0.951	0.970	0.991	0.552	0.741	0.742	0.507	0.702	0.760
80	0.968	0.946	0.992	0.573	0.155	0.539	0.564	0.119	0.473
90	0.950	0.778	0.961	0.686	0.070	0.274	0.685	0.111	0.082

Linear regression between the contraction (CD)/calcium transient durations (CTD) at percentages of the maximum peak height (10, 25, 50, 80 and 90) for each pair of measured signals

#### Calcium-Motion Coupling

The concurrent calcium-contraction measurement allows the measurement of synchronicity in calcium and motion dynamics. Here, we looked at the temporal differences between these signals by calculating the time points of maximum rate of change in the onsets and offsets of calcium transient and motion from Ca (bright) and CaPIV, respectively. The obtained values are shown in Table 3.

**Table 3:** Time intervals between calcium and contraction.

	WT	CPVTa	CPVTb
Ca (bright) and CaPIV difference on onset	163 ± 132 ms	238 ± 136 ms	126 ± 102 ms
Ca (bright) and CaPIV difference on offset	40 ± 91 ms	24 ± 142 ms	113 ± 75 ms

Mean time differences and standard deviations between the calcium transient and contraction in different cell lines, in milliseconds. The differences between cell lines are not statistically significant

Positive time values in onset and offset indicate that motion signal is following the calcium transient. Some negative values in offset were obtained indicating that motion reached its peak relaxation velocity before maximum calcium decline velocity. In Table 3 this is indicated by large standard deviation compared to the offset value especially for CPVTa. Although CPVTa shows a higher onset and CPVTb a higher offset in mean times, the findings are not statistically significant due to the large variability. Also these results reveal that CPVTa and CPVTb show different contraction-calcium coupling dynamics from WT but also different from each other.

The difference between the means of motion and calcium signal parameters was statistically significant in WT in all parameters (*p* < 0.05 in CD/CTD10, *p* < 0.01 in the remaining parameters), but not in CPVTa or CPVTb. This finding shows that measurements of motion and calcium provide complementary information and insight on the Ca^2+^ and contraction coupling dynamics.

When comparing the cell lines, the motion modalities PIV and CaPIV did not exhibit significant differences between the contraction durations of WT and CPVT cell lines. However, calcium modalities Ca (bright) and Ca (dark) showed significant differences between means of WT and CPVTb in CTD80 and CTD90. Neither calcium nor motion modalities displayed significant differences between WT and CPVTa.

## Discussion

We developed and demonstrated a method for simultaneous CM motion and calcium transient recording from fluorescent videos. The method uses simultaneous application of both transmission and fluorescent light source. In traditional methods the calcium based fluorescent signal could represent a source of error for PIV analysis and further, the brightfield imaging could affect the calcium imaging transients. We tested the method susceptibility to both error sources by separating these factors with interlacing brightfield and fluorescent video frames and observed that a simultaneous measurement of calcium and contraction is feasible.

We recorded consecutive frames of brightfield imaging and calcium fluorescent imaging in iPSC-CMs on video, with background light on and off. Four different signals were obtained: motion from brightfield video (PIV), motion from video with fluorescence (CaPIV), calcium imaging without background light (Ca (dark)) and calcium imaging with background light (Ca (bright)). We characterized the signals for both validation and demonstration of the method. With this procedure, we aimed to demonstrate that CM contraction could be quantified reliably while running a simultaneous fluorescent measurement.

Further, we demonstrated the usefulness of the simultaneous measurement by comparing the calcium and contraction signals, calculating time intervals between calcium and motion, and analyzing the differences between WT and two different CPVT cell lines.

### Effect of Fluorescent Light on Motion Analysis

Previously, the intensity changes caused by fluorescence have prevented simultaneous motion measurements. MQD, however, does not put emphasis on bright pixels thus making it a plausible method for the task. Our results show that the intensity fluctuation effect by fluorescent light is negligible to both timing and magnitude of the motion signal. This is the first time such measurement and assessment of accuracy have been done without using corrections with a fluorescent dye.

Our results indicate that MQD based analysis of CM motion is minimally affected by the presence of a fluorescence based measurement. In t-tests, there were no significant differences between the means of measured CD parameters. The *R* ^2^ values in linear regression studies between normal brightfield PIV and CaPIV with fluorescence were very high in all three cell lines indicating the very low impact of fluorescence presence. There were small differences in the lower parts of the peaks as well as a minor 5% difference in magnitudes of contraction velocities. We believe this measurement setup allows measuring of motion with a variety of different fluorescent indicators, as long as movement is present. Determining a sufficient level of lighting for the measurements may be a key factor in the acquisition of two simultaneous high quality recordings.

### Effect of Bright Light on Calcium Imaging

Applying transmission light had only small effect on the calcium imaging waveforms. In linear regression analysis, high *R* ^2^ values were obtained between Ca (bright) and Ca (dark), in all three cell lines. However, in CPVT some lower regression values were obtained due to a number of possible factors: calcium transients Ca (bright) and Ca (dark) were not from the same beats, the number of recorded cells in CPVT lines was low and as the beating rate was lower, the number of beats in each signal was smaller than in WT. As illustrated in the CPVTa signals shown in Fig. 2, the calcium transients could have multiple small peaks (i.e. arrhythmias/abnormal transients) making the direct comparison of peak width parameters difficult. For WT cells, the peaks were more regular and Ca (bright) and Ca (dark) were both capable of characterizing the cell lines similarly, as shown in t-tests. Ca (dark) and Ca (bright) amplitudes were not compared, as the comparison would not have been valid due to the bleaching of fluorescent dye during the measurement. Moreover, it is evident that increased background light increases the background noise level in fluorescence measurement. However, sufficient transmission light or constant fluorescent light is needed for detection of motion signal from the video. At the same time, the fluorescent emission of the dye should be strong enough to be detected while the transmission video is acquired. Increasing the light intensity or exposure time of the sample may not be the best solutions since they result in higher photobleaching of fluorophores and slower frame rates.

Ratiometric dyes have different emission spectra for bound and unbound states with the indicated ion, for example with Fura-2. Therefore, it takes two consecutive channel recordings to capture the ratio between these states, which takes longer and results in lower frame rates in acquisition. In this case, as an additional channel for brightfield video is measured, non-ratiometric dyes, such as Fluo-4, are better since their emission needs to be recorded only on one wavelength rather than ratiometric dyes. The results indicate that in calcium studies, with a focus on the waveforms instead of absolute Ca^2+^ concentration, it is feasible to apply also the brightfield light to obtain additional information on the mechanical activity without affecting the calcium measurement.

### Cell Line Differences

There was no statistically significant differences in *t*-tests between motion in WT and CPVT cell lines when considering PIV or CaPIV. CPVT cells typically present arrhythmias under adrenergic stimulation, but as only the baseline beating is analyzed here, the motion signals correspond to those of WT. These results would indicate that the CPVT cells do not directly exhibit beating phenotypes different from WT cells at baseline conditions. Earlier, using PIV we have found differing beating phenotypes in LQT cells, but of course also the clinical presentation of the diseases are different.^9^

Ca (bright) and Ca (dark) showed statistically significant differences between WT and CPVTb in CTD80 and CTD90, but not between WT and CPVTa. These differences could be caused by a higher variation in diastolic calcium levels in CPVTb caused by a leakage of calcium through RYRs in the cells. However, the amplitude of these diastolic events is so low compared to the actual calcium peak so there were no differences in the parameters associated with the higher parts of the peak (i.e. CTD10).

The combination of motion and calcium measurements showed a varying disconnection of the two, as shown by the *R* ^2^ values (Table 2) especially for CPVTa. The most prevalent cause for CPVT lies in mutations in RYR2 gene coding for RYR.^20^ It is located at the membrane of sarcoplasmic reticulum in CMs and acts as a channel releasing calcium from sarcoplasmic reticulum to cytosol. Mutations in RYR2 cause spontaneous calcium leakage through the channel and altered calcium transients inside CMs.^13^ In CPVTb population, which carries a V4653F mutation in RYR, the substituted amino acid is located in the channel domain of the protein.^16^ It is thought to be a gain-of-function point mutation, as most of the RYR mutations, and alter the channels sensitivity or permeability to calcium ions.^23^ CPVTa population, which carries an exon 3 deletion, has the structural defect on the cytosolic part of the protein in the N-terminal domain and is associated with more severe clinical outcome.^12^ The difference in the nature of these two mutations could explain the differences in our results. The disconnection between calcium and beating motion could be related to the earlier finding by Tang* et al*. where the exon 3 deletion was found to be resulting in an abnormal termination of calcium release through the RYR.^24^

### Simultaneous Calcium and Motion Measurements

The combined simultaneous recording of motion and calcium as shown here provides new tools for studying the CM contraction mechanism. In t-tests between motion and calcium modalities, statistical differences were seen in WT cell lines, but not in CPVTa/CPVTb. The lack of statistical difference between motion and calcium in CPVT was surprising and it may again be caused by the low number of recorded cells or more heterogeneous beating patterns and calcium transients. In linear regression analysis of motion and calcium modalities, represented in Table 2, CPVTa had lower *R* ^2^ values than WT and CPVTb: there was practically no relation between the measurements in peak parameters characterizing the low amplitudes.

Mean time differences of signal onset and offset rate of change maxima did not show statistically significant differences between the cell lines due to high variability and low number of cells measured. However, CPVTa did show indication of longer time intervals between calcium and motion in onset, and CPVTb in offset.

Overall, the results indicate that calcium and motion go hand in hand as is expected, but one cannot directly be deduced from the other. Although the low correlation values in CPTVa may be caused by the low number of samples, high correlation values were not present for WT either where the sample number was higher. This reinforces the idea that even if Ca^2+^ is the driving force in CM contraction, calcium measurement alone cannot be used to describe the mechanical function in full detail. Further, motion alone does not necessarily reflect the ionic functions, emphasizing the need for the combined method suggested here as a new tool for CM analysis.

### Method Advantages

Video-based measurements are minimally invasive and have low instrumentation requirements. These aspects make them a feasible candidate for high-throughput studies. For laboratories, this method can be used in conjunction with other studies, as microscopes and video cameras are mainstay laboratory equipment. The threshold for including contraction measurements in studies is lower than measurements with other methods, because no additional equipment is needed. Combining the method with fluorescent beads, substrate deformation and thus contraction force could be measured as well. While here we have considered individual cells and small clusters, the method should be applicable for larger samples of cardiac tissue, as long as 2-dimensional approach is still feasible. As we have shown here, the possibility of measuring CM contraction from calcium imaging data also enables revisiting previous studies and expanding on their results.

### Study Limitations and Sources of Error

To validate the method we used interlaced video. The time difference between PIV and CaPIV videos—by being consecutive frames and the relatively low frame rate—must be taken into account when estimating their differences and similarities. The frame interlacing produces intrinsic time differences of 21.7 and 8.8 ms for our minimum frame rate 46 and maximum frame rate 114, respectively. A higher frame rate with slow beating cells would give a more accurate reading. However, the observed differences between normal brightfield PIV and fluorescence CaPIV were small, indicating that MQD-based motion measurements are minimally obstructed by fluorescence signals. We have previously explored waveform averaging in brief,^2^ suggesting a frame rate of 60. Further studies with a higher frame rate and more beats per video would better quantify the difference in amplitudes for measurements where absolute precision of magnitude is required. Due to the low number of recordings, the findings related to the disconnection between calcium and motion in CPVT cells should be considered tentative. A study focusing on calcium-motion coupling in CPVT cells is required for reliability.

In this study, CMs were cultured on glass coverslips where their morphology is different from mature human CMs. Therefore, results may be different using i.e. patterned CMs. However, our method should be applicable to those studies also.

Our method uses simultaneous optimized transmission and fluorescent light sources. The results showed that the added light source had very small effect on the detected fluorescent signal. However, the method is producing additional light load to the fluorescent dye and cells and thus photobleaching of the dye and phototoxicity should be considered. As our method uses no fluorescent signal background correction, it is straightforward to compute and free from possible correction errors.

## Conclusion

We have developed and evaluated a protocol for simultaneous calcium and motion signal measurement. By measuring the same motion from videos with and without fluorescence, we were able to validate and quantify the accuracy of a simultaneous measurement. Our results show that MQD based PIV is minimally affected by fluorescence. Furthermore, by measuring the calcium transients with and without background light, we showed that simultaneous brightfield imaging causes minimal interference to the calcium measurement. This is the first time simultaneous measurements of video-based CM motion with fluorescent dye based calcium transients are conducted without fluorescent video background corrections. It opens a new avenue to study the simultaneous ionic and biomechanical functions and the differences in their dynamics.

Our demonstration of simultaneous measurement implies that combined imaging is applicable also with other fluorescent reporters. Combining the measurement of motion would provide an additional layer of information for fluorescence studies. All these will open a new door to greater understanding of electromechanical coupling in CM contraction, disease mechanisms and drug effects.

## Electronic supplementary material

Below is the link to the electronic supplementary material.

Supplementary material 1 (DOCX 14 kb)

## Abbreviations

CaPIV: Calcium particle image velocimetry
CD: Contraction duration
CM: Cardiomyocyte
CPVT: Catecholaminergic polymorphic ventricular tachycardia
CTD: Calcium transient duration
hiPSC: Human induced pluripotent stem cell
iPSC: Induced pluripotent stem cell
iPSC-CM: Induced pluripotent stem cell derived cardiomyocyte
PIV: Particle image velocimetry
RYR: Cardiac ryanodine receptor
MQD: Minimum quadratic difference
WT: Wild type

## References

[1] Ahola A, Kiviaho AL, Larsson K, Honkanen M, Aalto-Setälä K, Hyttinen J (2014). Video image-based analysis of single human induced pluripotent stem cell derived cardiomyocyte beating dynamics using digital image correlation. Biomed. Eng. Online.

[2] Ahola, A., P. Pradhapan, E. Laurila, K. Aalto-setälä, and J. Hyttinen. Motion Analysis method for determining cardiomyocyte beating properties based on digital image correlation and templates. In Computing in Cardiology Conference (CinC), 2014, pp. 1137–1140, 2014.

[3] Bedut S, Seminatore-Nole C, Lamamy V, Caignard S, Boutin JA, Nosjean O, Stephan J-P, Coge F (2016). High-throughput drug profiling with voltage- and calcium-sensitive fluorescent probes in human iPSC-derived cardiomyocytes. Am. J. Physiol. Heart Circ. Physiol..

[4] Grespan E, Martewicz S, Serena E, Le Houerou V, Rühe J, Elvassore N (2016). Analysis of calcium transients and uniaxial contraction force in single human embryonic stem cell-derived cardiomyocytes on microstructured elastic substrate with spatially controlled surface chemistries. Langmuir.

[5] Gui LC, Merzkirch W (1996). A method of tracking ensembles of particle images. Exp. Fluids.

[6] Hayakawa T, Kunihiro T, Dowaki S, Uno H, Matsui E, Uchida M, Kobayashi S, Yasuda A, Shimizu T, Okano T (2012). Noninvasive evaluation of contractile behavior of cardiomyocyte monolayers based on motion vector analysis. Tissue Eng. Part C Methods.

[7] Huebsch N, Loskill P, Mandegar MA, Marks NC, Sheehan AS, Ma Z, Mathur A, Nguyen TN, Yoo JC, Judge LM, Spencer CI, Chukka AC, Russell CR, So P-L, Conklin BR, Healy KE (2015). Automated video-based analysis of contractility and calcium flux in human-induced pluripotent stem cell-derived cardiomyocytes cultured over different spatial scales. Tissue Eng. Part C. Methods.

[8] Hwang HS, Kryshtal DO, Feaster TK, Sánchez-Freire V, Zhang J, Kamp TJ, Hong CC, Wu JC, Knollmann BC (2015). Comparable calcium handling of human iPSC-derived cardiomyocytes generated by multiple laboratories. J. Mol. Cell. Cardiol..

[9] Kiviaho AL, Ahola A, Larsson K, Penttinen K, Swan H, Pekkanen-Mattila M, Venäläinen H, Paavola K, Hyttinen J, Aalto-Setälä K (2015). Distinct electrophysiological and mechanical beating phenotypes of long QT syndrome type 1-specific cardiomyocytes carrying different mutations. IJC Hear. Vasc..

[10] Kujala K, Paavola J, Lahti A, Larsson K, Pekkanen-Mattila M, Viitasalo M, Lahtinen AM, Toivonen L, Kontula K, Swan H, Laine M, Silvennoinen O, Aalto-Setala K (2012). Cell model of catecholaminergic polymorphic ventricular tachycardia reveals early and delayed afterdepolarizations. PLoS ONE.

[11] Lahti AL, Kujala VJ, Chapman H, Koivisto A-P, Pekkanen-Mattila M, Kerkelä E, Hyttinen J, Kontula K, Swan H, Conklin BR, Yamanaka S, Silvennoinen O, Aalto-Setälä K (2012). Model for long QT syndrome type 2 using human iPS cells demonstrates arrhythmogenic characteristics in cell culture. Dis. Models Mech..

[12] Lobo PA, Kimlicka L, Tung C-C, Van Petegem F (2011). The deletion of exon 3 in the cardiac ryanodine receptor is rescued by beta strand switching. Structure.

[13] MacLennan DH, Chen SRW (2009). Store overload-induced Ca(2+) release as a triggering mechanism for CPVT and MH episodes caused by mutations in RYR and CASQ genes. J. Physiol..

[14] Maddah M, Heidmann JD, Mandegar MA, Walker CD, Bolouki S, Conklin BR, Loewke KE (2015). A non-invasive platform for functional characterization of stem-cell-derived cardiomyocytes with applications in cardiotoxicity testing. Stem Cell Rep..

[15] Paci, M., E. Passini, S. Severi, J. Hyttinen, and B. Rodriguez. A population of in silico models to face the variability of human induced pluripotent stem cell-derived cardiomyocytes: The hERG block case study. In Computing in Cardiology Conference (CinC), pp. 1189–1192, 2016.

[16] Peng W, Shen H, Wu J, Guo W, Pan X, Wang X, Chen SRW, Yan N (2016). Structural basis for the gating mechanism of the type 2 ryanodine receptor RyR2. Science.

[17] Penttinen K, Siirtola H, Àvalos-Salguero J, Vainio T, Juhola M, Aalto-Setälä K (2015). Novel analysis software for detecting and classifying Ca^2+^ transient abnormalities in stem cell-derived cardiomyocytes. PLoS ONE.

[18] Penttinen K, Swan H, Vanninen S, Paavola J, Lahtinen AM, Kontula K, Aalto-Setälä K (2015). Antiarrhythmic effects of dantrolene in patients with catecholaminergic polymorphic ventricular tachycardia and replication of the responses using iPSC models. PLoS ONE.

[19] Pesl M, Pribyl J, Acimovic I, Vilotic A, Jelinkova S, Salykin A, Lacampagne A, Dvorak P, Meli AC, Skladal P, Rotrekl V (2016). Atomic force microscopy combined with human pluripotent stem cell derived cardiomyocytes for biomechanical sensing. Biosens. Bioelectron..

[20] Priori SG, Napolitano C, Tiso N, Memmi M, Vignati G, Bloise R, Sorrentino V, Danieli GA (2001). Mutations in the cardiac ryanodine receptor gene (hRyR2) underlie catecholaminergic polymorphic ventricular tachycardia. Circulation.

[21] Ribeiro MC, Tertoolen LG, Guadix JA, Bellin M, Kosmidis G, D’Aniello C, Monshouwer-Kloots J, Goumans M-J, Wang Y, Feinberg AW, Mummery CL, Passier R (2015). Functional maturation of human pluripotent stem cell derived cardiomyocytes *in vitro*—Correlation between contraction force and electrophysiology. Biomaterials.

[22] Rodriguez AG, Han SJ, Regnier M, Sniadecki NJ (2011). Substrate stiffness increases twitch power of neonatal cardiomyocytes in correlation with changes in myofibril structure and intracellular calcium. Biophys. J..

[23] Roston TM, Van Petegem F, Sanatani S (2017). Catecholaminergic polymorphic ventricular tachycardia: a model for genotype-specific therapy. Curr. Opin. Cardiol..

[24] Tang Y, Tian X, Wang R, Fill M, Chen SRW (2012). Abnormal termination of Ca^2+^ release is a common defect of RyR2 mutations associated with cardiomyopathies. Circ. Res..

