# Supplementary material for: Simultaneous Measurement of Contraction and Calcium Transients in Stem Cell Derived Cardiomyocytes
Source: Ann Biomed Eng. 2017 Oct 3;46(1):148–58. doi: 10.1007/s10439-017-1933-2 (PMC5754453; doi:10.1007/s10439-017-1933-2)
Supplement: Supplementary file 1 — Supplementary material 1 (DOCX 14 kb) [file 10439_2017_1933_MOESM1_ESM.docx]

Human iPSC culturing and cardiomyocyte differentiation

Human iPSC lines were established by sendai viral (CytoTune® iPS reprogramming kit, Thermo Fisher Scientific, Waltham, MA, USA) or retroviral transfection of *OCT3/4*, *SOX2*, *KLF4* and *c-MYC* ^3^. Characterization of hiPSC lines was done as described by Lahti et al ^1^ and Penttinen et al ^4^.

Differentiation into CMs was carried out by either co-culturing hiPSCs with murine visceral endoderm-like (END-2) cells (prof. Mummery, Hubrecht Institute, Utrecht, The Netherlands) ^2^. After 15 days of co-culturing, hiPSCs formed spontaneously beating clusters, which were cut and dissociated with collagenase A (Roche Diagnostics, Basel, Switzerland). Cells were plated on round 12 mm coverslips.

Imaging protocol

Cells were loaded with 4 μM Fluo-4 AM (Molecular Probes, Life Technologies Ltd) for 30 minutes and de-esterified for 10 minutes at 37 °C in perfusion medium: (in mM) 137 NaCl, 5 KCl, 0.44 KH2PO4, 20 HEPES, 4.2 NaHCO3, 5 D-glucose, 2 CaCl2, 1.2 MgCl2 and 1 Na-pyruvate dissolved in H2O. pH of the perfusion medium was adjusted to 7.4 with NaOH and temperature controlled at 37°C with an inline heater (Warner Instruments, Hamden, CT, USA) during perfusion. Combined calcium and video imaging was conducted with Olympus IX71 inverted microscope using UApo/340 x20 NA0.75 air objective (Olympus, Tokyo, Japan). For video acquisition, ANDOR iXon 885 EMCCD camera (Andor Technology, Belfast, Northern Ireland) was used without binning orEM-gain and synchronized with a Polychrome V light source by a real time DPS control unit. Fluo-4 AM was excited at 488nm and for brightfield video acquisition Olympus TH4-200 light source was used without phase contrast method. Fluorescent and bright field signals were recorded through Olympus U-MF2 Alexa 488 bandpass filter cube (ex.470-495, em.525/50 nm). LiveAcquisition software (TILL Photonics, Munich, Germany) was used to control Polychrome V light source and camera during recording.

For both calcium and video acquisition 5 ms excitation times were used to record two channels consecutively. During the recording, the Olympus light source was turned on to obtain video image while still being able to discern calcium fluctuations. The recording process is illustrated in Figure 1A. Cropping of active camera chip and minimal cycle times were used resulting in total frame rates of 46-114 (23-57 frames per second per channel) in recordings. In total, 25 videos were recorded: 7 CPVTa, 5 CPVTb and 13 WT videos. The recordings were all baseline measurements.

1. Lahti, A. L., V. J. Kujala, H. Chapman, A.-P. Koivisto, M. Pekkanen-Mattila, E. Kerkelä, J. Hyttinen, K. Kontula, H. Swan, B. R. Conklin, S. Yamanaka, O. Silvennoinen, and K. Aalto-Setälä. Model for long QT syndrome type 2 using human iPS cells demonstrates arrhythmogenic characteristics in cell culture. *Dis. Model. Mech.* 5:220–230, 2012.
2. Mummery, C., D. Ward-van Oostwaard, P. Doevendans, R. Spijker, S. van den Brink, R. Hassink, M. van der Heyden, T. Opthof, M. Pera, A. B. de la Riviere, R. Passier, and L.Tertoolen. Differentiation of human embryonic stem cells to cardiomyocytes: role of coculture with visceral endoderm-like cells. *Circulation* 107:2733–2740, 2003.
3. Ohnuki, M., K. Takahashi, and S. Yamanaka. Generation and characterization of human induced pluripotent stem cells. *Curr. Protoc. Stem Cell Biol.* Chapter 4:Unit 4A.2, 2009.
4. Penttinen, K., H. Swan, S. Vanninen, J. Paavola, A. M. Lahtinen, K. Kontula, and K. Aalto-Setälä. Antiarrhythmic Effects of Dantrolene in Patients with Catecholaminergic Polymorphic Ventricular Tachycardia and Replication of the Responses Using iPSC Models. *PLoS One* 10:e0125366, 2015.
